# Bacterial coinfections in COVID-19-hospitalized patients

**DOI:** 10.1590/1806-9282.2023469

**Published:** 2024-03-04

**Authors:** Ingrid Stéfanie Sarmento Debaco, Helena Moreira Kluck, Rômulo Marx, Paulo Ricardo Mottin da Rosa, Cassiano Teixeira

**Affiliations:** 1 Our Lady of Conception Hospital, Conceição Hospital Group, Internal Medicine Service – Porto Alegre (RS), Brazil.; 2 Moinhos de Vento Hospital, Internal Medicine Service – Porto Alegre (RS), Brazil.; 3 Universidade Federal de Ciências da Saúde de Porto Alegre, Department of Clinical Medicine and Rehabilitation Sciences – Porto Alegre (RS), Brazil.

**Keywords:** COVID-19, Coinfection, Bacteria, Internal medicine, Pseudomonas infections

## Abstract

**OBJECTIVE:**

The aim of this study was to assess the rate of bacterial infections in COVID-19-hospitalized patients and to analyze the most prevalent germs, sources, risk factors, and its impact on in-hospital mortality.

**METHODS:**

This observational retrospective study was conducted on 672 patients hospitalized between April and August 2020 in Nossa Senhora da Conceição Hospital, a public hospital located in Porto Alegre, Brazil. The inclusion criterion was adult patients hospitalized with confirmed COVID-19. Data were collected through chart review. Risk factors for bacterial infection and mortality were analyzed using both univariate and multivariate robust Poisson regression models.

**RESULTS:**

Bacterial coinfection was observed in 22.2% of patients. Risk factors for bacterial infections were dementia (RR=2.06 (1.18–3.60); p=0.011), cerebrovascular disease (RR=1.75 (1.15–2.67); p=0.009), active cancer (RR=1.52 (1.082–2.15); p=0.01), need for noninvasive ventilation (RR=2.320 (1.740–3.094); p<0.01), invasive mechanical ventilation (RR=4.63 (2.24–9.56); p<0.01), and renal replacement therapy (RR=1.68 (1.26–2.25); p<0.01). In the adjusted model, bacterial infections were not associated with mortality (0.96 (0.75–1.24); p=0.79). The most common source of infection was due to respiratory, blood, and central venous catheters, with 69 (29.36%), 61 (25.96%), and 59 (25.11%) positive cultures, respectively.

**CONCLUSION:**

We observed a high rate of bacterial infections in COVID-19-hospitalized patients, most commonly of respiratory source. Neurologic and oncologic morbidities and need for ventilation and renal replacement therapy was associated with risk factors for bacterial infections. Nevertheless, an association between bacterial infections and hospital mortality was not established.

## INTRODUCTION

In December 2019, the initial cases of pneumonia caused by a novel coronavirus (2019-nCoV) were identified in the city of Wuhan, China^1,2^. Then, SARS-CoV-2 (severe acute respiratory syndrome coronavirus 2) rapidly spread, resulting in a global pandemic. As of the completion of this study, a total of 5,451,900 deaths from the disease have been reported worldwide^3^. A report from the Chinese Center for Disease Control classified the severity of the disease as follows: mild (absent or mild pneumonia: 81% of cases), severe (dyspnea, hypoxemia, or pulmonary involvement greater than 50%: 14% of cases), and critical (respiratory failure, shock, or multiple organ dysfunction: 5% of cases)^4^. Regarding the Brazilian context, the study published by Ranzani et al.^5^ analyzed the first 250,000 hospitalizations for the disease in the country, showing an overall hospital mortality rate of 38%. Mortality in patients transferred to the ICU was 59%, and in patients undergoing mechanical ventilation (MV), it was 80%. Mortality in the state of Rio Grande do Sul, where the present study was conducted, was 31%.

There are still relatively few published studies on bacterial complications in COVID-19-hospitalized patients^6-8^. In these studies, rates of bacterial infections vary between 19%^9^ and 3.6%^10^. The most common pathogens include *Streptococcus pneumoniae*, *Staphylococcus aureus*, *Pseudomonas aeruginosa*, and *Escherichia coli*. However, there is a lack of published data related to our specific environment.

This was an observational retrospective study conducted at a Brazilian public tertiary hospital. The objective was to assess the rates of nosocomial infectious complications, defined as microbiological evidence emerging after 48 h of hospitalization in COVID-19 patients, admitted to Hospital Nossa Senhora da Conceição during the SARS-CoV-2 pandemic. The study aimed to evaluate the most prevalent pathogens and infection sites, risk factors for infectious complications, and their impact on mortality.

## METHODS

This study was approved by the Ethics Committee of the Grupo Hospitalar Conceição on December 8, 2021, with the registration number 31719920.8.0000.5530.

We conducted a retrospective cohort study on hospitalized patients with COVID-19 from April 2020 to August 2020. The patients were consecutively included during the study period. Demographic, laboratory, and microbiological data were obtained through electronic medical record review. The inclusion criterion was adults hospitalized with COVID-19 in the study period. Patients included had SARS-CoV-2-confirmed infection according to the hospital infection committee. Confirmatory tests were as follows: antigen, polymerase chain reaction, or GeneXpert molecular test. The tests were used to confirm infection varied during the study period due to resource limitations imposed by the pandemic.

The study was conducted at Hospital Nossa Senhora da Conceição, a federal hospital that is part of the Grupo Hospitalar Conceição Hospital Complex. It is a public and teaching hospital with 875 clinical and surgical beds. It is a national reference for treating hospitalized patients with SARS-CoV-2 infection during the pandemic.

Variables analyzed were as follows: age, gender, color, length of stay, HIV infection, chronic kidney disease, dementia, cerebrovascular disease, systemic arterial hypertension, chronic lung disease, rheumatologic disease, chronic heart disease, active cancer, cirrhosis, diabetes mellitus, obesity, blood test, bilirubin, lactic dehydrogenase, dyspnea at admission, altered consciousness at admission, hemoptysis at admission, oxygen use, NIV and IMV, vasopressor use, ICU admission, need for RRT, and chest X-ray abnormalities. Blood cultures, urine cultures, and sputum cultures were analyzed according to their positivity and the isolated germ.

Only cases with microbiological evidence were considered bacterial infections. Thus, blood cultures, catheter blood cultures, urine cultures, and tracheal aspirates were analyzed. Cases in which only one blood culture was positive for known contaminants and the attending team had not initiated antimicrobial treatment were not considered bacterial complications. Associated with the culture positivity, there had to be a diagnosis of a bacterial infection by the assisting team.

### Statistical analysis

Quantitative variables, depending on their nature, were analyzed using mean and standard deviation or median and interquartile range. Other variables were analyzed through their relative and absolute frequencies. To evaluate the risk factors for bacterial complications, we performed a robust Poisson regression. In the univariate analysis, variables with p<0.1 were considered eligible for the multivariate model. In the multivariate model, variables with p<0.05 were considered significant. A multivariate Poisson regression model was also performed to evaluate the risk factors for mortality, using bacterial complications as the independent variable.

For this analysis, we used Microsoft Excel 2010 and the R software (R Core Team (2021). R: A language and environment for statistical computing. R Foundation for Statistical Computing, Vienna, Austria) and Rstudio (RStudio Team [2021]. RStudio: Integrated Development Environment for R. RStudio, PBC, Boston, MA).

## RESULTS

The demographic characteristics and clinical laboratory aspects of the patients are detailed in Table 1. The prevalence of nosocomial bacterial infections in conjunction with SARS-CoV-2 infection stood at 22.2%. Among the 672 patients under analysis, there were 171 deaths, resulting in a mortality rate of 25.4%. In the model constructed with eligible variables for multivariate analysis (p<0.1) concerning the occurrence of bacterial infection, the following variables exhibited statistical significance: dementia, cerebrovascular disease, active cancer, NIV, IMV, and the necessity for hemodialysis. The relative risks and corresponding confidence intervals from both univariate and multivariate Poisson analyses are presented in Table 2.

**Table 1.** Characteristics of the 672 patients included in the study.

Variables	n
Age [mean (standard deviation)]	59.00 (17.17)
Race (%)	
White	531 (79.0)
Indigenous	1 (0.1)
Black	106 (15.8)
Pardo	34 (5.1)
Male (%)	348 (51.8)
Length of stay (median [IQR])	11.0 [5.0, 23.0]
HIV (%)	19 (2.8)
CKD (%)	38 (5.7)
Dementia (%)	36 (5.4)
Cerebrovascular disease (%)	64 (9.5)
Systemic arterial hypertension (%)	306 (45.5)
Chronic lung disease (%)	94 (14.0)
Rheumatologic disease (%)	13 (1.9)
Chronic heart disease (%)	124 (18.5)
Active cancer (%)	99 (14.7)
Cirrhosis (%)	4 (0.6)
Diabetes mellitus (%)	171 (25.4)
Obesity (%)	84 (12.5)
Neutrophils (median [IQR])	71.5 [62.8, 78.2]
Lymphocytes (median [IQR])	15.6 [9.4, 22.9]
C-reactive protein (median [IQR])	93.4 [44.4, 169.5]
BD (median [IQR])	0.18 [0.11, 0.32]
LDH (median [IQR])	551.0 [382.5, 738.5]
Dyspnea at admission (%)	510 (76.3)
Altered consciousness at admission (%)	78 (11.6)
Hemoptysis at admission (%)	5 (0.7)
Oxygen use (%)	544 (81.2)
Noninvasive mechanical ventilation (%)	37 (5.5)
Invasive mechanical ventilation (%)	227 (33.9)
Vasopressor use (%)	210 (31.6)
ICU admission (%)	235 (35.0)
Renal replacement therapy (%)	74 (11.0)
Chest X-ray abnormalities (%)	540 (80.5)
Outcomes	
Death (%)	171 (25.4)
Bacterial infections (%)	149 (22.2)

CKD: chronic kidney disease; CRP: C-reactive protein; DB: direct bilirubin; LDH: lactate dehydrogenase; ICU: intensive care unit; IQR: interquartile range.

**Table 2.** Poisson model with robust variance for the outcome bacterial infection.

Variables	Model 1		Model 2	
	RR (95%CI)	p	RR (95%CI)	p
Age	1.01 (1.00–1.02)	0.004	1.00 (0.99–1.01)	0.92
Dementia	1.54 (0.95–2.51)	0.078	2.06 (1.18–3.60)	0.01
Cerebrovascular disease	1.73 (1.20–2.49)	0.003	1.75 (1.15–2.67)	0.009
Hypertension	1.38 (1.04–1.84)	0.024	1.09 (0.82–1.46)	0.53
Chronic heart disease	1.40 (1.02–1.94)	0.036	1.02 (0.73–1.42)	0.89
Active cancer	1.39 (0.99–1.97)	0.057	1.52 (1.08–2.15)	0.01
Altered consciousness	1.69 (1.20–2.38)	0.003	1.33 (0.89–1.98)	0.15
Oxygen use	2.40 (1.40–4.10)	0.001	0.74 (0.40–1.37)	0.34
NIV	3.32 (2.50–4.42)	<0.01	2.32 (1.74–3.09)	<0.01
Invasive MV	4.60 (3.36–6.30)	<0.01	4.63 (2.24–9.56)	<0.01
Use of vasopressors	3.87 (2.88–5.19)	<0.01	0.80 (0.41–1.57)	0.53
RRT	3.49 (2.71–4.50)	<0.01	1.68 (1.26–2.25)	<0.01

NIV: non-invasive mechanical ventilation; IMV: invasive mechanical ventilation; VAD: vasoactive drugs; RRT: renal replacement therapy. Model 1=univariate analysis. Model 2=regression adjusted for the variables included in the model.

The same model was employed in multivariate Poisson regression to assess the risk factors associated with mortality, considering bacterial infections as an independent variable. The use of oxygen during hospitalization was excluded from the model, given that all patients who succumbed to the disease used oxygen. This adjustment was deemed unnecessary and could introduce potential bias in relation to the mortality outcome. Age, active cancer, altered consciousness, IMV, and requirement for RRT emerged as statistically significant variables for the analyzed outcome. Bacterial infections (adjusted RR=0.94 (95%CI 0.73–1.20)) did not exhibit a statistically significant association with the examined outcome. The data pertaining to the mortality outcome are provided in Table 3.

**Table 3.** Poisson model with robust variance for the outcome of death.

Variables	RR (95%CI)	p
Age	1.03 (1.02–1.04)	<0.01
Bacterial infection	0.96 (0.75–1.24)	0.79
Dementia	1.16 (0.67–2.02)	0.58
Cerebrovascular disease	1.06 (0.75–1.48)	0.72
Hypertension	1.01 (0.79–1.29)	0.92
Chronic heart disease	1.18 (0.94–1.49)	0.14
Active cancer	1.89 (1.42–2.52)	<0.01
Altered consciousness	1.34 (1.00–1.80)	0.04
NIV	0.71 (0.43–1.16)	0.18
IMV	3.08 (1.70–5.58)	<0.01
VAD	1.62 (0.92–2.86)	0.09
RRT	1.62 (1.29–2.03)	<0.01

NIV: non-invasive mechanical ventilation; IMV: invasive mechanical ventilation; VAD: vasoactive drugs; RRT: renal replacement therapy.

Details regarding the bacteria isolated in the analyzed cultures and the affected sites can be found in Table 1. The most prevalent sources of infection were respiratory, blood, and central venous catheters, accounting for 69 (29.36%), 61 (25.96%), and 59 (25.11%) positive cultures, respectively. *Pseudomonas aeruginosa* was the most frequently identified respiratory pathogen.

## DISCUSSION

In our cohort of 672 patients hospitalized for COVID-19 in a tertiary hospital in southern Brazil, we observed a bacterial infection rate concurrent with SARS-CoV-2 infection of 22.2%. Our findings indicated higher rates compared to those reported in other studies. Among the 235 positive cultures, the most frequent infection sites were the respiratory tract, with 69 cases (29.36%), and peripheral blood cultures, with 61 cases (25.95%). When excluding coagulase-negative staphylococci (CNS) from blood cultures, this rate decreased to 32 cases (13.61%), placing it below urinary tract infections (19.57%).

Until now, the SARS-CoV-2 pandemic in Brazil has yielded a total of 22,328,252 cases. Considering that approximately 14% of patients are at risk of developing severe cases and 5% may progress to critical illness, the pandemic has strained both the Brazilian public and private healthcare systems, posing a threat to their sustainability, as these patients require significant hospital support. Hospitalized patients face an elevated risk of developing infectious complications, which significantly affects overall patient outcomes and escalates the complexity and cost of care. Jie Li et al.^6^ in their analysis of 1,495 cases of COVID-19 hospitalization in Wuhan, demonstrated a high risk of bloodstream infections in critically ill patients. In our study, patients at a higher risk of developing infectious complications, after adjusting for other variables, included those with dementia, cerebrovascular disease, and neoplasia. Patients requiring NIV, IMV, and RRT also exhibited an increased risk of developing infectious complications. Neto et al.^9^ reported a bacterial coinfection rate of 19% in a single-center study, with bacterial coinfection emerging as an independent risk factor for mortality. In a study conducted at the Hospital Clinic of Barcelona by Carolina Garcia-Vidal and colleagues^8^, a coinfection rate of 7.3% was observed, and patients with coinfections displayed higher rates of corticosteroid use, ICU admission, and chronic kidney disease. Hughes et al.^7^ documented bacterial coinfections in 6.1% of cases. A study conducted at the Union Hospital of Wuhan found that 6.8% of patients experienced bacterial coinfections, with half of these patients ultimately succumbing to the illness, underscoring the predictive power of bacterial complications for hospital mortality.

In our study, after adjusting for confounding factors, bacterial complications did not emerge as predictors of mortality. Several factors may explain this finding: given the retrospective nature of the study, some colonization cases may have been considered infections. Consequently, stable patients with positive cultures were grouped with those having bloodstream infections such as *Acinetobacter baumannii*. Another consideration is that unmeasured variables may have influenced the outcomes. Additionally, some infectious complications may not have been confirmed through culture, representing a limitation associated with laboratory data.

One factor contributing to the elevated rate of bacterial complications in our cohort is the clinical severity of the patients: the rate of ICU admission was 35%, and the need for NIV and IMV stood at 33.9%. In the study by Neto et al.^9^ patients exhibited IMV rates of 17% in the control group and 43% in the group with infectious complications. Mortality rates were 15% in the control group and 46% in the group with bacterial coinfections. Compared to the Brazilian study by Ranzani et al.^5^ which evaluated the first 250,000 COVID-19 hospitalizations in the country, our IMV rate was higher at 33.9% compared to the 23% rate found in the state of Rio Grande do Sul (our state). Despite this, our mortality rate was low: while Alvaro’s study reported a mortality rate of 38% in the country and 31% in our state, our cohort observed a mortality rate of 25.4%.

The sources of infection in our study were similar to those reported in other studies. The research conducted by Carolina Garcia-Vidal in Barcelona^8^ revealed that the primary infectious foci were the respiratory and bloodstream systems, with *Streptococcus pneumoniae*, *Pseudomonas aeruginosa*, and *Escherichia coli* being the most common pathogens. Hughes et al.^7^ documented low rates of coinfections, with the most common sources being respiratory, involving isolates of *Klebsiella pneumoniae* and *Enterobacter cloacae*. Jie Li et al.^6^ also identified the respiratory tract as the primary site of infection, with *Acinetobacter baumannii* and *Klebsiella pneumoniae* being the predominant isolates. Alvaro Neto and colleagues^9^ noted that the urinary tract was the most frequent focus. In our study, the focus of infection was primarily the respiratory system, with the most common isolates being *Pseudomonas aeruginosa*, *Enterobacter* sp., and *Klebsiella pneumoniae*. The second most common focus was the bloodstream; however, 47.54% of these cases were likely due to contaminant organisms.

Our study has several limitations, primarily its observational and retrospective nature, which restricted data collection and certain inferences. Consequently, we were unable to clearly distinguish between patients with colonization and those with infections caused by the identified pathogens in cultures. A prospective analysis with well-defined criteria would have increased the reliability of our evaluation. Nonetheless, our study possesses several strengths. To the best of our knowledge, it represents the first analysis of bacterial coinfections in COVID-19-hospitalized patients conducted in Brazil. Furthermore, it was conducted at the largest public hospital in southern Brazil and served as a national reference in the battle against the pandemic.

## CONCLUSION

In our investigation involving a cohort of COVID-19-confirmed patients admitted to a tertiary Brazilian hospital, we observed a heightened incidence of bacterial coinfections in comparison with prior research findings. Predominantly, the respiratory tract and bloodstream constituted the primary sites of infection. Nevertheless, we did not identify a significant correlation between bacterial infections and mortality in this patient population.
